# Endoscopic Ultrasonography‐guided Variceal Therapy as Salvage Treatment for Rebleeding From Duodenal Varices Following Balloon‐occluded Retrograde Transvenous Obliteration

**DOI:** 10.1002/deo2.70183

**Published:** 2025-08-14

**Authors:** Sakue Masuda, Atsushi Irisawa, Soichiro Nakaya, Jun Kubota, Karen Kimura, Makomo Makazu, Kazuya Koizumi

**Affiliations:** ^1^ Department of Gastroenterology Medicine Center Shonan Kamakura General Hospital Kanagawa Japan; ^2^ Department of Gastroenterology Dokkyo Medical University School of Medicine Tochigi Japan

**Keywords:** balloon‐occluded retrograde transvenous obliteration, cyanoacrylate injection, duodenal varices, endoscopic ultrasound, variceal bleeding

## Abstract

Duodenal varices, though rare, are potentially life‐threatening complications of portal hypertension. Management is challenging when balloon‐occluded retrograde transvenous obliteration (BRTO) fails to achieve complete obliteration. Endoscopic ultrasonography‐guided variceal therapy (EUS‐VT) is effective for gastric varices, but reports of duodenal varices remain limited. We present a case of a 71‐year‐old woman with alcoholic liver cirrhosis and duodenal variceal bleeding. Initial hemostasis was achieved with endoscopic variceal ligation, followed by BRTO. However, rebleeding occurred due to incomplete obliteration caused by dual afferent veins. EUS‐VT comprising n‐butyl cyanoacrylate was performed as salvage therapy because of ascites and vascular complexity. Despite technical challenges, EUS‐VT successfully obliterated the varices. Post‐procedural computed tomography showed lipiodol migration into the portal system. No rebleeding or liver dysfunction occurred. Ascites worsened—likely because of BRTO and/or EUS‐VT—but was manageable with low‐dose diuretics. This case highlights EUS‐VT as a feasible option after failed BRTO for duodenal varices. A meticulous technique is essential to preventing glue migration.

## 1 Introduction

Esophageal, gastric, and duodenal varices are major complications of portal hypertension. Duodenal varices represent only 1% to 3% of cases, but they are associated with a high risk of fatal bleeding [1, 2]. Bleeding from duodenal varices is associated with a mortality rate up to 40% which is often attributable to liver failure after hemostatic treatment and a 9% recurrence rate. Rebleeding often occurs within 6 weeks [2]. Thus, close monitoring and elective treatments such as balloon‐occluded retrograde transvenous obliteration (BRTO) or endoscopic therapy are recommended even after initial hemostasis.

Endoscopic ultrasonography (EUS) provides high‐resolution imaging of vasculature and has been shown to effectively visualize gastric varices [3, 4, 5]. However, only five reports have described its use as a first‐line approach for duodenal varices, and no prior reports have evaluated EUS‐guided variceal therapy (EUS‐VT) as salvage therapy after BRTO [3, 6]. Few of these reports have described variceal size; however, larger lesions tend to be treated with adjunctive coil embolization. Additionally, an inconsistent n‐butyl cyanoacrylate (NBCA)–lipiodol dilution ratio ranging from 50% to 100% has been reported. We present a case of rebleeding following BRTO with incomplete obliteration that was treated using EUS‐VT with NBCA as salvage therapy.

## 2 Case Presentation

**FIGURE 1:**
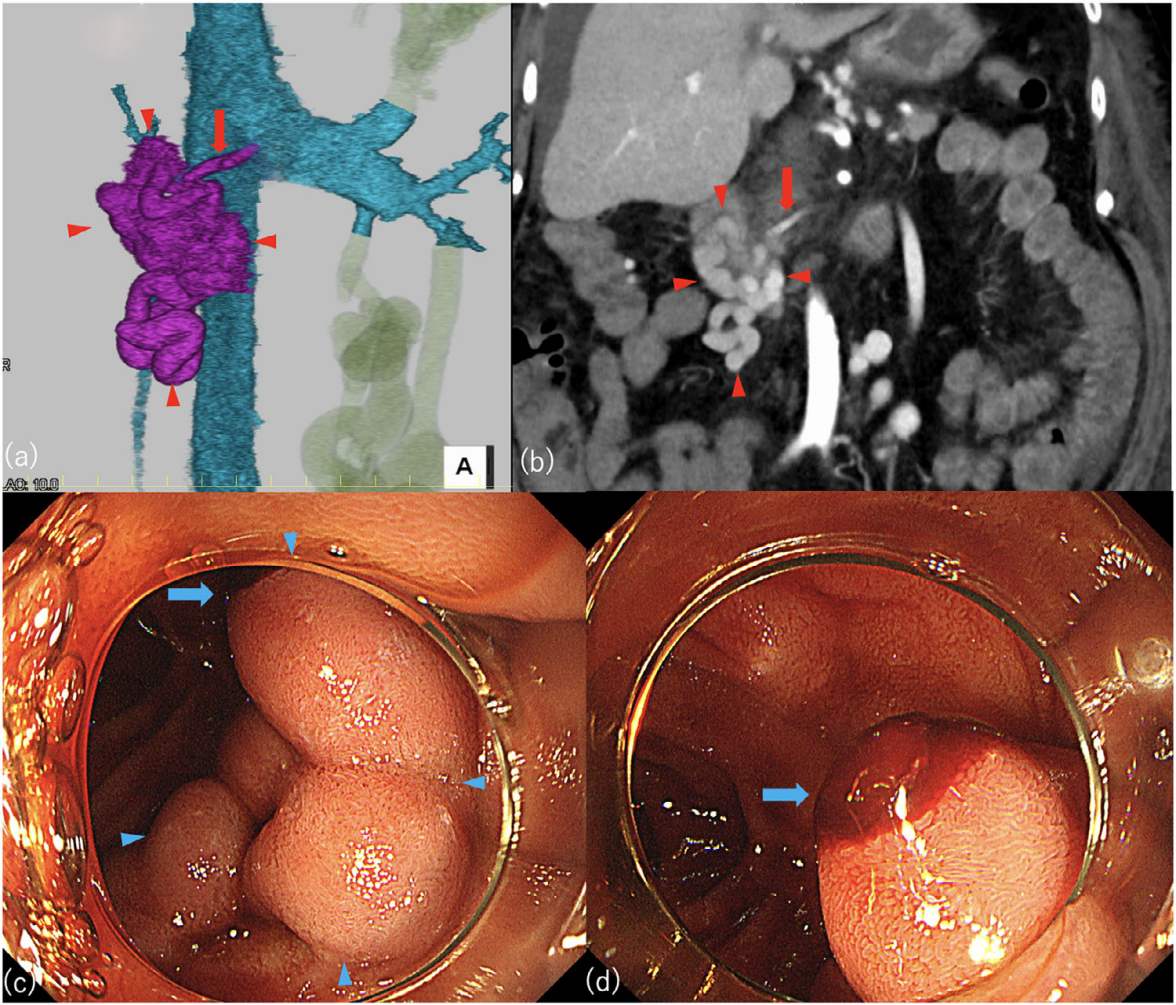
Contrast‐enhanced computed tomography images of duodenal varices and pre‐treatment endoscopic images of duodenal varices. (a) Three‐dimensional reconstructed image. (b) Coronal section. (c) Endoscopic view showing all the duodenal varices. (d) Endoscopic close‐up of the rupture site. Red arrowhead: duodenal varices. Red arrow: feeder vein originating from the proximal superior mesenteric vein. Blue arrowhead: duodenal varices. Blue arrow: rupture site.

**FIGURE 2:**
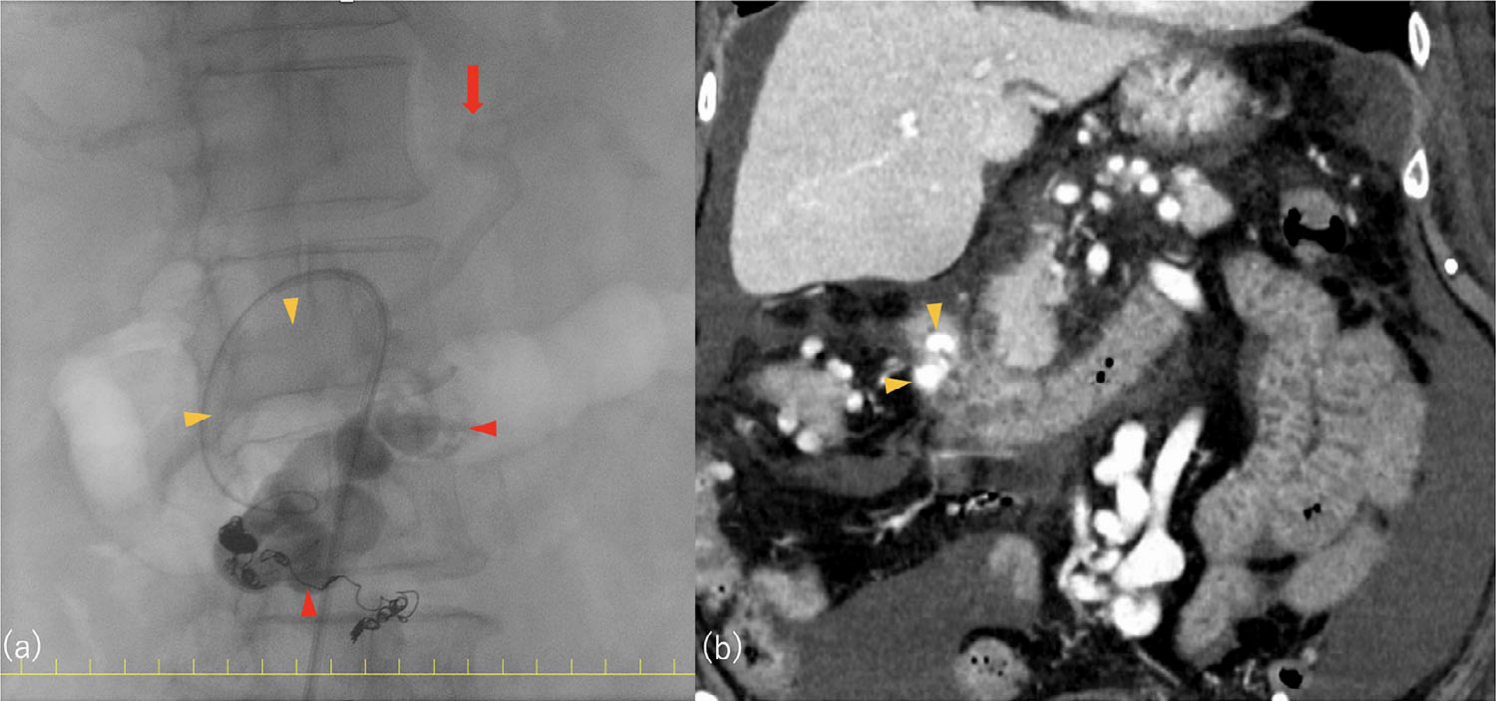
Partial obliteration of duodenal varices following balloon‐occluded retrograde transvenous obliteration (BRTO) that resulted in incomplete embolization of the distal superior mesenteric vein (SMV) feeder area. (a) Fluoroscopic images acquired during BRTO. (b) Contrast‐enhanced computed tomography (CT) image obtained 10 days after BRTO. Yellow arrowhead: region of duodenal varices not obliterated by BRTO. Red arrowhead: region of duodenal varices successfully obliterated by BRTO. Red arrow: feeder vein originating from the proximal superior mesenteric vein.

A 71‐year‐old woman with alcoholic liver cirrhosis (Child‐Pugh class B) was referred for breast cancer treatment. Duodenal varices were incidentally discovered during chemotherapy. Four months later, variceal bleeding occurred (Figure 1). Emergency endoscopic variceal ligation (EVL) achieved hemostasis and was followed by BRTO. Contrast‐enhanced computed tomography (CT) revealed two feeder veins arising from the proximal and distal superior mesenteric veins (SMVs) and one efferent vein draining into the ovarian vein. BRTO obliterated most of the varices and the proximal feeder but failed to fully embolize the distal feeder and part of the varices (Figure 2). Ten days later, rebleeding from the residual segment occurred. Emergency EVL was performed as a bridging procedure because a hemodynamic assessment was necessary. Based on the CT findings, EUS‐VT was selected instead of percutaneous transhepatic obliteration and endoscopic injection sclerotherapy (EIS) because of the presence of ascites and anatomical proximity to the feeder. Compared to conventional EIS, EUS‐VT enables more reliable puncture of the varix in close proximity to the feeder vein.

**FIGURE 3:**
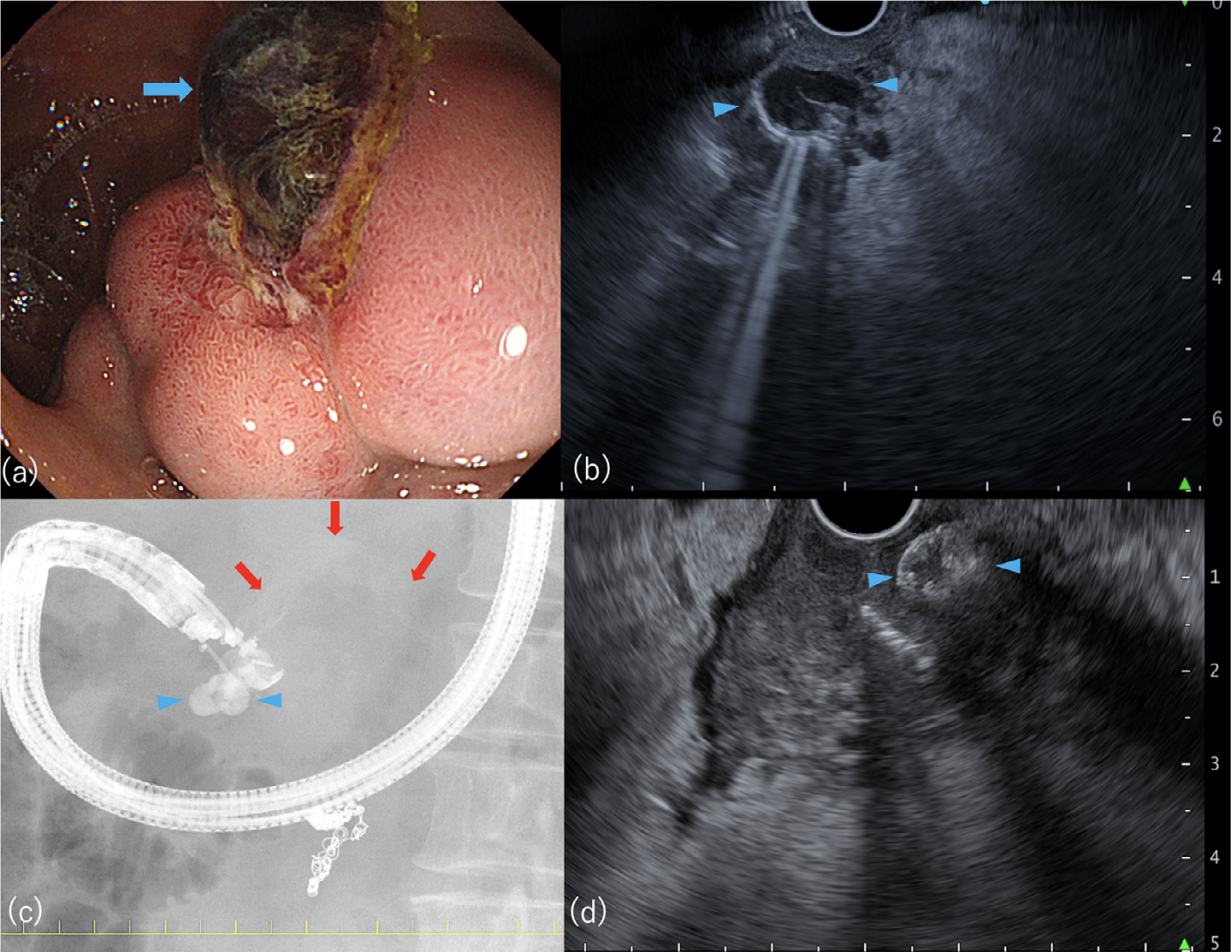
Endoscopic and endoscopic ultrasonography (EUS) images at the time of rebleeding. (a) Rupture site identified using endoscopy. (b) Duodenal varix visualized on EUS (short axis, 5 mm). (c) N‐butyl cyanoacrylate (NBCA) injected during EUS‐guided variceal therapy (EUS‐VT). (d) Injected NBCA visualized on EUS. Blue arrow: rupture site of the duodenal varix. Blue arrowhead: duodenal varix. Red arrow: feeder vein originating from the distal superior mesenteric vein.

EUS‐VT was performed using a convex‐arrayed echoendoscope (GF‐UCT260; Olympus Corp., Tokyo, Japan) the following day. The O‐ring from the prior EVL had detached, and the varices were ulcerated. A 5‐mm lesion was punctured using a 22‐gauge fine‐needle aspiration needle (Sonotip ProControl; Medi‐Globe, Oberbayern, Germany) under EUS guidance. Needle placement was confirmed by blood backflow and contrast injection. After flushing with 5% dextrose, NBCA was injected; however, premature solidification occurred, necessitating a second puncture. A prefilled extension tube comprising a mixture of NBCA and lipiodol (1.5 mL + 0.5 mL) was administered, and flushing with 5% dextrose was performed. Although the estimated dead space was 5 mL (3.9 mL in the extension tube and approximately 1 mL in the needle), 6.5 mL of dextrose was used for high‐pressure flushing to prevent occlusion. EUS and fluoroscopy confirmed adequate glue distribution and complete obliteration (Figure 3).

**FIGURE 4:**
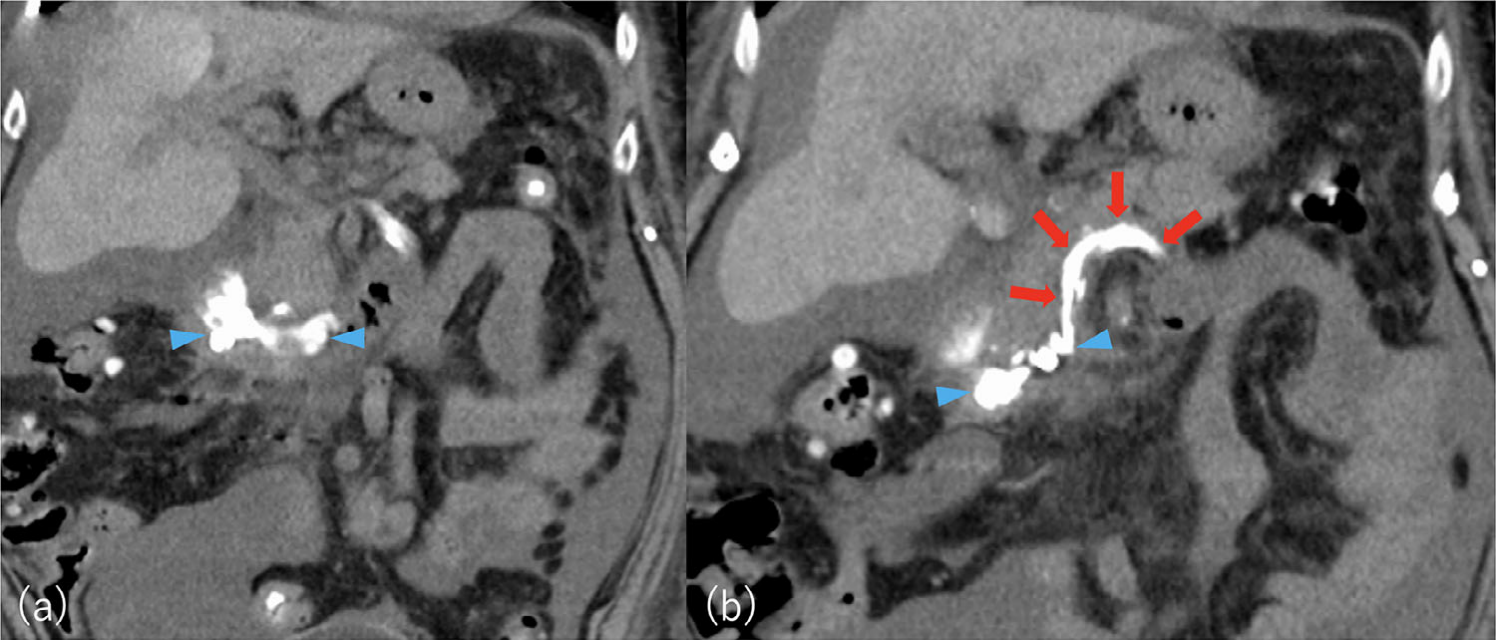
Computed tomography (CT) image after endoscopic ultrasonography (EUS)‐guided variceal therapy. (a) *N*‐butyl cyanoacrylate (NBCA) distributed within duodenal varices. (b) NBCA distributed within the duodenal varices and feeder vein originating from the distal superior mesenteric vein (SMV), the SMV, and the splenic vein. Blue arrowhead: NBCA distributed within the duodenal varices. Red arrow: NBCA distributed within the feeder vein (originating from the distal SMV), the SMV, and the splenic vein.

Post‐procedural CT revealed glue migration into the SMV, splenic vein, and inferior mesenteric vein (Figure 4). However, no hepatic dysfunction or hyperammonemia was observed. A CT evaluation performed 1 month later confirmed the persistence of NBCA in the varix and decreased distribution in the portal vein. Ascites, which had been present before treatment, worsened after BRTO and EUS‐VT but improved with low‐dose diuretics. No rebleeding occurred during the 1‐month follow‐up period.

## 3 Discussion

Reports of EUS‐VT for duodenal varices are extremely limited [3, 7]. This case demonstrates the utility of EUS‐VT as salvage therapy after incomplete obliteration by BRTO. Initial bleeding was controlled with emergency EVL followed by BRTO to prevent recurrence. However, bleeding recurred as a result of dual feeder veins and required a second EVL. The efferent vein had already been occluded; therefore, repeat BRTO was technically difficult. Because of the presence of ascites, we avoided percutaneous approaches and selected EUS‐VT as a minimally invasive option, which effectively obliterated the residual varices and feeders. The presence of dual feeders emphasized the complexity and potential value of EUS‐VT when standard methods are inadequate.

Conventional EIS has a technical success rate of approximately 95% but is associated with complication and rebleeding rates of 30% and 23%–40%, respectively [8]. Compared to EIS, EUS‐VT offers similar technical success and, possibly, fewer complications [9]. Nevertheless, reports remain scarce [6], and EUS‐VT is not widely adopted, typically requiring institutional review board approval [5], as in our case.

EUS offers several advantages over traditional methods [3], such as precise sizing and localization of varices, identification of feeders or shunts, real‐time needle guidance, Doppler‐based confirmation of obliteration, and reduced reliance on endoscopic visualization during active bleeding or poor visibility. However, because of the elasticity and mobility of veins, EUS‐VT may require technical skills comparable to or exceeding those required for EUS hepaticogastrostomy. In our case, EUS allowed clear visualization of varices and an anatomically favorable puncture path, thus enabling precise NBCA delivery. Doppler imaging was not useful, likely because of decreased flow after BRTO.

Premature NBCA solidification at the needle tip necessitated a second puncture. To prevent this, a preloaded extension tube comprising NBCA was administered, and flushing with 5% dextrose was performed. Although 5 mL of dextrose was initially calculated from the device volumes, 6.5 mL was used to ensure full flushing and prevent blockage. The NBCA volume should be adjusted based on the variceal size. Previous studies of gastric varices recommended 2–4 mL of NBCA [3]. Organ embolism is a serious adverse event associated with volume [6]. Flushing carries the risk of premature polymerization during the brief interval required to switch syringes from NBCA to 5% dextrose, which may result in needle lumen obstruction. In addition, excessive volumes of dextrose may increase the risk of unintended glue migration into non‐target vessels. Therefore, we suggest avoiding dextrose flushing during EUS‐VT for small duodenal varices. Instead, we recommend calculating the full required volume, including the dead space, and administering the injection under fluoroscopic guidance using a single step.

The NBCA concentration used during EUS‐VT is not standardized and varies from 50% to 100% [6, 10]. Higher concentrations may lower the embolism risk but impair visibility. We chose an NCBA concentration of 75% for this case. Prior to BRTO, which resulted in shrinkage of the varices and occlusion of the main efferent vein, along with excessive flushing and a low NBCA concentration, likely contributed to NBCA migration into the superior mesenteric and splenic veins. Migration was not detected in real time, suggesting it progressed in a diluted form that was not visible on fluoroscopy [6]. A lower proportion of lipiodol in the NBCA mixture reduces radiopacity; therefore, visualizing the real‐time distribution of glue during fluoroscopy and detecting unintended migration after the procedure are more difficult. Although firm conclusions cannot be drawn from only one case, a higher NBCA concentration may have reduced the migration risk.

This study had limitations. Technical success was assessed using plain CT, which showed lipiodol distribution. NBCA remained in the varices after the procedure, but the cast glue precluded a short‐term size comparison. Follow‐up CT and endoscopy at 6 months after the procedure were scheduled; however, at the time of reporting, follow‐up CT was performed only 1 month after the procedure had been performed. No rebleeding was observed, but this post‐procedural period was too short to assess long‐term recurrence and the rebleeding risk. These limitations should be considered when evaluating the utility of EUS‐VT.

Despite these issues, this case adds to the limited evidence supporting EUS‐VT as salvage therapy for complex cases. Furthermore, this case demonstrates the feasibility of EUS‐VT and highlights the need for care when using NBCA in small varices. Although EUS‐VT was effective for this case, it should be considered as part of a multidisciplinary approach rather than a stand‐alone solution. EUS‐VT may serve as a valuable, yet previously underutilized, treatment option for complex cases of duodenal varices; however, further cases and long‐term outcomes should be evaluated to confirm its safety and efficacy.

## Ethics Statement

All procedures were performed in accordance with the ethical standards of the 1964 Declaration of Helsinki and its later amendments.

## Consent

Informed written consent was obtained from the patient for the performance of EUS‐VT.

## Conflicts of Interest

The authors declare no conflicts of interest.

## Data Availability

The technical appendix, statistical code, and dataset are available from the corresponding author upon request. No additional data are available for this study.
